# Development of Acellular Matrix-Based Bioprinted Scaffold for Inferior Alveolar Nerve Regeneration

**DOI:** 10.1021/acsomega.6c01059

**Published:** 2026-06-11

**Authors:** Nasera Rizwana, Kaustubh Raundal, Yogesh H S, Radhika Kawathe, Sridhar Chinthakindi, Rohit Kumar Sarda, Ashwath Acharya, Manasa Nune

**Affiliations:** a Manipal Institute of Regenerative Medicine, 76793 Manipal Academy of Higher Education, Manipal, Karnataka 576104, India; b Department of Pharmacology, NITTE College of Pharmaceutical Sciences (NITTE Deemed to be University), Bangalore Campus, Karnataka 560064,India; c 76793 Manipal Academy of Higher Education, Manipal, Karnataka 576104, India; d 29126 Institute of Bioinformatics Discoverer Building, seventh Floor, International Tech Park, Whitefield, Bangalore, Karnataka 560 066, India; ⊥ Department of Anatomy, Sikkim Manipal Institute of Medical Sciences, Sikkim Manipal University, Tadong, Gangtok, Sikkim 737102, India; # Department of Hand Surgery,, 29224 Kasturba Medical College, Manipal, Centre for Congenital Hand Differences, Manipal, Karnataka 576104, India

**Keywords:** inferior alveolar nerve, peripheral nerve regeneration, bioprinting, cellular matrix, alginate, methylcellulose

## Abstract

The inferior alveolar nerve (IAN) is a sensory branch of the mandibular nerve that supplies sensation to teeth, the chin, and the lower lip. IAN injuries often result from trauma or iatrogenic causes, leading to the development of symptoms like difficulty in eating, smiling, pain, and paraesthesia. IAN regeneration is limited because of the confinement of the nerve within the mandibular canal. Current pharmacological and surgical interventions offer only partial recovery. Therefore, alternative approaches to enhance nerve repair and regeneration are required. To address this limitation, we developed a 3D-bioprinted scaffold composed of alginate, methylcellulose, and Schwann cell-derived acellular matrix (Alg/MC/ACM) for IAN regeneration. The Alg/MC hydrogel showed good mechanical stability and printability, and ACM provides biological cues for nerve regeneration. Proteomics analysis showed that there were 904 common proteins present when the Schwann cell lysate was compared with ACM. The rheological analysis demonstrated shear-thinning and viscoelastic properties of the Alg/MC/ACM hydrogel. In vitro cytocompatibility tests, such as MTT and Live/Dead assays using rat Schwann (RSC96) cells and neuronal (PC12) cells, demonstrated that Alg/MC/ACM2 significantly enhanced cell proliferation and viability when compared to Alg/MC and Alg/MC/ACM1 scaffolds. In vivo study in SD rat IAN crush injury model demonstrated that implantation of Alg/MC/ACM2 scaffolds improved the behavioral response when compared with injury control. Alg/MC/ACM2 scaffolds also promoted cellular infiltration, axonal organization, and remyelination, as observed in hematoxylin and eosin and Luxol fast blue staining. Overall, the incorporation of Schwann-cell-derived ACM within Alg/MC scaffolds provided a bioactive microenvironment that supported neural regeneration. This study presents the first evidence demonstrating the potential of Alg/MC/ACM bioprinted scaffolds as promising therapeutic strategies for IAN repair.

## Graphical abstract

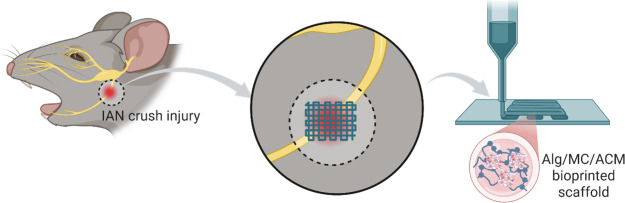

## 1 Introduction

The inferior alveolar nerve (IAN) is a sensory branch of the mandibular nerve that supplies sensation to the lower teeth, chin, and lower lip. It traverses the mandibular canal, branching into the mental and incisive nerve in the lower lip and chin area, making it more susceptible to iatrogenic injuries during dental and maxillofacial procedures (Figure 1). Due to the close proximity of the mandibular (lower) third molar tooth to the IAN, there is undoubtedly a risk of injury to the nerve during surgical management of impacted third molars or during orthognathic surgeries.^1–5^ Injury to the IAN is also one of the most common consequences in the oral and maxillofacial region during accidents. Other causes of IAN injury are nontraumatic injuries such as head and neck tumors, which add to the IAN injury burden in India.^6^

**Figure 1:**
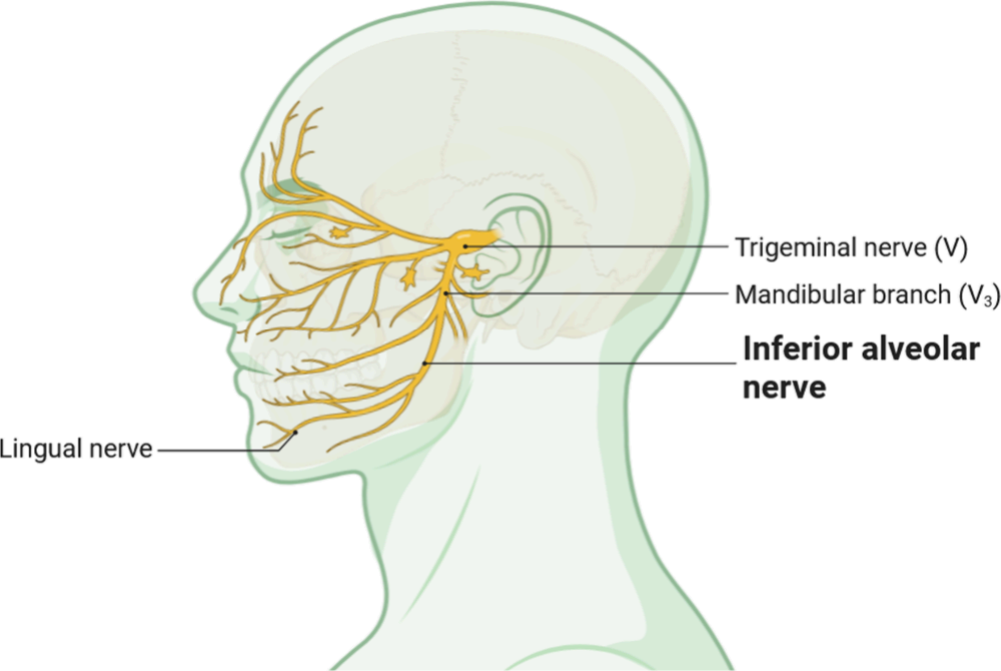
Schematic representation of the course of inferior alveolar nerve. Created using BioRender.com.

Among the different categories of peripheral nerve injuries described by Seddon and Sunderland, neuropraxia or crush injury of the IAN is relatively common.^7,8^ IAN is often compressed or contused without complete transection. Crush injuries are known to spontaneously regenerate owing to the innate property of the peripheral nerve to regenerate. Crush injury induces a characteristic pattern of axonal degeneration and demyelination known as Wallerian degeneration distal to the site of insult. The mechanical trauma leads to disruption of axonal continuity, altered ion channel permeability, cytoskeletal breakdown, and calcium influx that initiates axonal degeneration cascades. Schwann cells distal to the lesion undergo dedifferentiation, proliferate, and begin phagocytosis of myelin debris, while macrophages infiltrate to facilitate debris clearance and release proregenerative cytokines. However, the regenerative process is complex and highly time sensitive. Fibrosis and glial scar formation can impede axonal regrowth.^9^ In particular, with IAN injury, regeneration is further limited as it is enclosed in a bony canal with restricted space and compromised vasculature. This delays the infiltration of Schwann cells and macrophages, and the diffusion of neurotrophic factors, leading to slow regeneration. The majority of injuries lead to hypersensitivity of teeth, pain, paraesthesia, hypothesia, drooling of saliva, involuntary biting of soft tissues, and limitations in speaking, eating, and drinking, leading to a decrease in the quality of life of a patient.^10–12^ Postinjury fibrosis or chronic compression further impairs reinnervation. Clinical observations have shown that even after months, many patients continue to experience residual sensory deficits, emphasizing the need for interventions that can accelerate and organize nerve repair. In a study done in South India, the overall prevalence of IAN injury was 56.3%. Minimal changes or improvement in the symptoms were seen for 1 week. At 6 months, 23.9% of cases still had some form of IAN dysfunction, emphasizing the limitation of peripheral nerve regeneration.^13^

Clinically, the management of IAN injuries remains a significant challenge. The choice of treatment depends on the severity and type of injury. In mild neuropraxic cases, observation and pharmacologic management with drugs such as corticosteroids, nonsteroidal anti-inflammatory drugs, vitamin B complexes, or neurotrophic agents are known to facilitate spontaneous recovery.^6,14–16^ When crush injuries are severe, conservative treatment approaches fail to restore nerve function. In cases where crush injuries persist for a long duration or nerve discontinuity is present, microsurgical repair techniques such as neurorrhaphy, grafting, or nerve conduits are employed to promote axonal regeneration. However, the enclosed anatomical pathway of IAN within the mandibular canal limits surgical accessibility. A study done by Bruce Donoff et al. stated that intervention to treat IAN injury delayed beyond the critical window of 3−6 months after injury significantly reduces the prognosis of the treatment.^17,18^ Previous studies have investigated growth factor−based approaches for inferior alveolar nerve regeneration. For example, Lee et al.^19^ developed an NGF-supplying dental implant in a canine IAN transection model, which demonstrated improved nerve conduction velocity, axon density, and myelin thickness compared with controls. Similarly, systemic administration of nerve growth factor in a rabbit distraction osteogenesis model was reported by Hergt et al.^20^ to enhance axonal regeneration and increase the density of myelinated fibers during IAN recovery. However, these approaches mainly relied on direct growth factor delivery, which may be limited by the short half-life, rapid diffusion, and lack of sustained localized release of bioactive molecules.^21,22^ Therefore, scaffold-based delivery systems capable of providing structural support along with controlled release of regenerative cues remain an effective treatment for IAN crush injuries, which have not been explored.

To this end, we hypothesized the development of 3D-bioprinted scaffolds for the treatment of IAN injury. Among the effective biomaterials used in bioprinting, alginate and methylcellulose have been widely used owing to their good biocompatibility, tunable mechanical properties, and ability to mimic the native extracellular matrix (ECM). Alginate is a naturally derived polysaccharide obtained from brown algae, which undergoes ionic cross-linking with divalent cations to form hydrogels. It provides a supportive microenvironment conducive to neural regeneration. However, its poor rheological properties and reduced cell adhesion necessitate blending with other polymers. Methylcellulose is a temperature-responsive and water-soluble cellulose derivative that is incorporated to improve the viscosity, printability, and mechanical integrity of the hydrogels. Together, the Alg/MC composite provides an optimal framework that ensures printability and shape fidelity required for peripheral nerve repair applications, as explored in our previous work. To further enhance the bioactivity of the scaffolds, a Schwann-cell-derived acellular matrix was incorporated within the Alg/MC hydrogel. ACM, as mentioned in our previous work, retains essential proteins such as S100, PMP22, and MPZ, which play an important role in peripheral nerve regeneration.^23^

In the present study, we investigate for the first time the development of a bioprinted scaffold composed of Schwann cell-derived acellular matrix blended within Alg/MC hydrogel, followed by a two-step cross-linking using calcium chloride for inferior alveolar nerve regeneration. Optimization of Alg/MC hydrogel, cross-linking for bioprinting, and thorough characterization of ACM were achieved in our previously published work.^24^ The Alg/MC/ACM hydrogel was optimized for rheological properties to ensure print fidelity and mechanical stability. By varying ACM concentration, the study aimed to assess its effect on Schwann (RSC96) and neuronal (PC12) cell viability and proliferation, while in vivo implantation in a rat IAN crush injury model assessed the scaffold’s regenerative potential through histological evaluation.

## 2 Methodology

### 2.1 ACM Preparation

ACM was prepared as described in our previously published work.^23^ Briefly, rat Schwann cells (RSC96) were cultured in a 100 mm dish in culture media consisting of DMEM, 10% FBS, penicillin-streptomycin, and antibiotic−antimycotic. After the cells had reached around 80−90% confluency, serum-free media was added and incubated for 24 h. Post 24 h, the media is discarded, and DI water was added and incubated for 2−3 min at room temperature. Once the swelling of cells is observed under a microscope, water is replaced with 0.1% ammonia solution for 5 min. Post 5 min, the ammonia solution is discarded, and the ACM thus obtained is washed with PBS and stored at −80 °C.

### 2.2 Proteomic Analysis

The sample preparation for proteomic analysis was performed as described in our previously published method.^25,26^ Briefly, ACM equivalent to 300 μg of total protein was aliquoted and adjusted to 200 μL with 100 mM TEAB (Triethylammonium bicarbonate, pH 8.5). Reduction and alkylation of proteins was conducted using DTT (dithiothreitol) and IAA (iodoacetamide). The proteins were digested by adding trypsin at a 10:1 (w/w) protein-to-enzyme ratio and incubating at 37 °C overnight. SDS-PAGE was performed to ensure complete protein digestion. Digestion was quenched with 20% trifluoroacetic acid, and the peptides were cleaned up using Sep-Pak C18 cartridges (Waters Corporation, Milford, MA, USA). The eluates were lyophilized and reconstituted in 0.1% formic acid prior to LC-MS/MS analysis.^25,26^ A similar procedure was adapted for Schwann cell lysate (CL) analysis.

A detailed LC-MS analysis and the instrumental parameters are provided in the SI. The acquired raw LC−MS/MS data files were processed using Proteome Discoverer software (version 2.4) with a label-free quantification workflow. Protein identification was performed using Sequest HT database integrated with software. The search was conducted against UniProt *Rattus norvegicus* protein sequences (accessed on 28 November 2025)*.* All searches required 10 ppm precursor mass tolerance, 20 ppm fragment mass tolerance, and fully tryptic cleavage specificity with 2 missed cleavages. Carbamidomethylation of cysteine was set as a static modification, while oxidation of methionine was a dynamic modification. The protein false discovery rate (FDR) was set at 1%. Only high-confidence peptide-spectrum matches (PSMs) were retained for further analysis.^25,26^

### 2.3 Alg/MC/ACM Hydrogel Preparation

Alg/MC hydrogel was prepared as described in previously published work.^24^ Briefly, 1 g of alginate was dissolved in 16 mL PBS and heated at a temperature of 45 °C at 400 rpm. After complete dissolution, 72 mg of calcium chloride in 4 mL of PBS was added until a homogeneous mixture was obtained. Then, the temperature was raised to 80 °C, and 1400 mg methyl cellulose was added and stirred at 1200 rpm. After complete dissolution, the temperature was reduced to 25 °C, and two concentrations of ACM (1 μg/mL and 10 μg/mL) were added and stirred at 200 rpm for 5 min. Hydrogel with 1 μg/mL of ACM was labeled as Alg/MC/ACM1, and hydrogel with 10 μg/mL of ACM was labeled as Alg/MC/ACM2.

### 2.4 3D Bioprinting and Rheological Analysis

The hydrogel was transferred to the printing cartridge gently without incorporation of air bubbles into the Cellink Inkredible+ bioprinter, and printing was carried out as mentioned in previously published work.^24^ A grid scaffold (10 × 10 mm) consisting of three layers was printed at a flow rate of 600 mm/min, layer height of 0.4 mm, and pressure of 79 kPa using a 25 G nozzle at room temperature. Post printing, the bioprinted 3D structure was stabilized by subjecting it to 4% calcium chloride solution for 2−3 min. The calcium chloride solution was discarded, and the 3D structure was washed with PBS for 3 times.

The rheological properties of Alg, Alg/MC, Alg/MC/ACM1, and Alg/MC/ACM2 hydrogels were evaluated using a rheometer (MCR302 Anton Paar). The dynamic and loss moduli of the hydrogels were measured in an oscillatory mode using a 25 mm parallel plate at room temperature. Amplitude sweeps and shear-thinning behavior were analyzed at a constant angular frequency of 10 rad/s.

### 2.5 Swelling and Degradation Analysis

The in vitro swelling property of the lyophilized scaffolds was studied. Samples were weighed (w_i_) and immersed in PBS and incubated at 37 °C. At various time points, the scaffolds were dabbed with tissue to remove excess PBS and weighed (w_f_) until saturation. The experiment was performed in triplicate, and the swelling ratio was calculated using eq 1.

$$\mathrm{Swelling}\;\mathrm{ratio}\;(\%)=\left(\frac{w_{f}-w_{i}}{w_{i}}\right)\times 100$$ 1

The degradation behavior of the lyophilized scaffolds was assessed by measuring the mass loss over a period. The lyophilized scaffolds were weighed (*w*_i_) and immersed in PBS and incubated at 37 °C. To measure the weight loss, samples were removed from the PBS and dabbed with tissue to remove excess PBS and weighed (*w*_f_) at various time points. The experiment was carried out in triplicate. The degradation rate (%) was calculated using eq 2.

$$\mathrm{Degradation}\;\mathrm{ratio}\;(\%)=(\frac{w_{i}-w_{f}}{w_{i}})\times 100$$ 2

### 2.6 Cell Viability and Proliferation

Cell proliferation studies of rat Schwann cells and PC12 cells on the lyophilized scaffolds were conducted using the MTT assay (HiMedia) as per the manufacturer’s protocol. Approximately 10,000 cells were seeded on the lyophilized scaffolds for 1, 5, 7, and 14 days. At various day points, the media was discarded, and 200 μL of MTT reagent (0.5 mg/mL) was added and incubated for 4 h. Post-incubation, 100 μL DMSO was added to dissolve the formazan crystals, and absorbance was measured at 570 nm using a multiplate reader (HH34000000, PerkinElmer (Ensight)). MTT protocol was also followed for the control scaffolds (scaffolds without cells), and the absorbance was subtracted from the sample absorbance to plot the graph. The graphs were plotted using GraphPad Prism software.

Cell viability studies were conducted using Live/Dead staining (Invitrogen, USA) as reported by us previously^9^ to determine the viability of cells within the bioprinted scaffolds. Briefly, 1 × 10^4^ cells/ml RSC96 cells were incorporated into Alg/MC/ACM2 hydrogel to form a bioink. Bioprinting was carried out as mentioned in Section 2.4. At day 5, the media was discarded, and the scaffolds were subjected to the addition of 1 μM calcein-AM and incubated for 20 min in the dark in a humidified incubator at 37 °C. Next, 2 μM ethidium homodimer was added to the scaffold and incubated for 10 min in the dark in a humidified incubator at 37 °C. Post-incubation, the scaffolds were observed and imaged using confocal fluorescence microscopy (Olympus F4000).

### 2.7 Animal Surgery and Implantation of Scaffolds

An in vivo study was carried out in a rat model after obtaining ethical clearance from the IAEC (IAEC/KMC/25/2024). Female Sprague−Dawley (SD) rats weighing 250−300 g were obtained for the study. The rats were anesthetized using intraperitoneal injection of 2% xylazine. The protocol for exposure of IAN in rats was adopted from a previously published literature (Figure 5A­(a,b)).^27,28^ Briefly, the skin near the lower part of the face was incised, the masseter muscle was exposed, and a small part of the mandibular bone was osteotomized to expose the IAN. After the nerve exposure, IAN was crushed by using mosquito forceps for 30 s. A bioprinted Alg/MC/ACM2 grid structure of dimension 1 × 1 × 1 mm was cut into 4 equal pieces, and one part was placed at the crushed area. The soft tissues and the skin were closed using Liquibond. The treated animals were allowed to recover from anesthesia in their own cages at room temperature and were maintained with a standard diet and water. At day point 21, behavior analysis was conducted. Animals were sacrificed using a high dose of thiopentone anesthesia, and the IAN was excised and stored in formalin to perform histological analysis.

### 2.8 Behavior Analysis

Mechanical sensitivity of the lower part of the face was assessed by modifying von Frey analysis^27^ on day 21 using brushes of different thicknesses (toothbrush and paintbrush). Animals were habituated to the testing environment for 10−15 min prior to assessment. Mechanical stimulation was applied to the control and treatment sides using a soft paintbrush (light tactile stimulus) and a toothbrush (firmer mechanical stimulus). Each stimulus was applied for 1−2 s and repeated three times with 30 s intervals. Behavioral responses were recorded based on withdrawal and eye closure behavior.

### 2.9 Histopathological Analysis

Animals were sacrificed on day 21, and nerve stumps were harvested and fixed in 10% formalin to preserve the tissue section. Tissue or nerve from the proximal and distal parts of the resected portion of the nerve was excised. The tissues were embedded in paraffin and sectioned for the histological analysis. Histological assessment included hematoxylin and eosin (H&E) staining (AMD Laboratories) to evaluate the tissue architecture and cellular changes within the harvested tissue. Next, Luxol Fast Blue stain (HiMedia) was carried out to stain the lipoproteins present on the surface of the myelin sheath. The images were observed under a phase contrast microscope (Nikon 80i), and the images were analyzed using ImageJ software.

### 2.10 Statistical Analysis

All the quantitative data of the results are presented as mean ± standard deviation (SD). The results were analyzed using two-way analysis of variance (ANOVA), followed by Tukey’s multiple comparisons test. Statistical analysis was conducted with at least 3 independent samples per experiment. A *p*-value of <0.05 was considered statistically significant.

## 3 Results and Discussion

### 3.1 Proteomic Analysis of ACM

**Figure 2:**
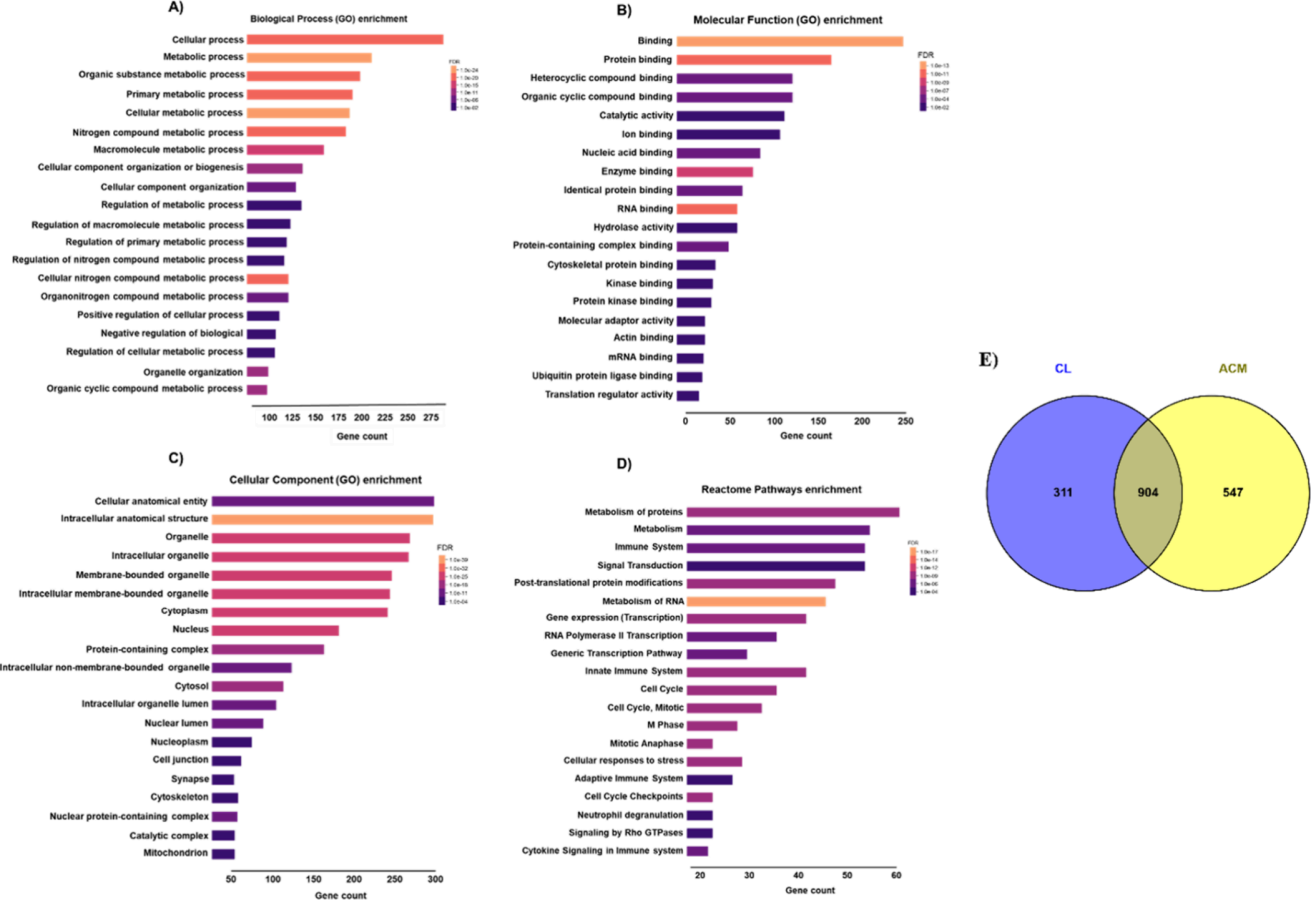
Functional clustering of identified proteins in Schwann cell decellularized matrix (ACM) and pathway analysis using STRING protein−protein interaction analysis. Gene ontology enrichment analysis of the (A) biological process, (B) molecular function, (C) cellular component, and (D) reactome pathway. Only the top 20 entries ranked with genes are shown here for vertical sizing. (E) Venn diagram showing the common and unique proteins between CL and ACM.

On the basis of the search criteria, we identified 1451 proteins in the ACM (Table S1). Proteomic analysis of cell lysate identified 4,132 peptides mapping to 1,215 proteins (Table S2). Comparative analysis using Venny 2.0 showed 904 proteins common to both CL and ACM, while 547 proteins were uniquely present in ACM (Figure 2E and Table S3).

To explore the functional roles of identified ACM proteins, we performed clustering using STRING protein−protein interaction (PPI) software (version 12.0) against the *Rattus norvegicus* genome database. The ACM-specific proteins were classified according to Gene Ontology (GO) categories: cellular components (CC), biological processes (BP), and molecular functions (MF), ranked by gene count (Figure 2, Table S4).^29^ According to the GO enrichment analysis of CC, proteins associated with the intracellular region, such as cellular and intracellular anatomical entity, membrane-bound organelle, cytoplasm, and nucleus, were highly enriched, consistent with the ACM preparation.^30^ According to GO enrichment analysis of MF, binding-related terms such as protein binding, nucleic acid binding, kinase binding, receptor binding, and ion binding are highly enriched, reflecting the ACM’s role in signaling and ligand−receptor interactions.^30^ Within BP, the enrichment of most of the metabolic processes, such as cellular, primary, nitrogen, and organic substances, and macromolecule compounds related to biological process highly enriched, indicating similar functions to the cellular matrix. Reactome pathway analysis revealed that the identified proteins were majorly involved in many of the cell-related functional pathways.^30^

**Figure 3:**
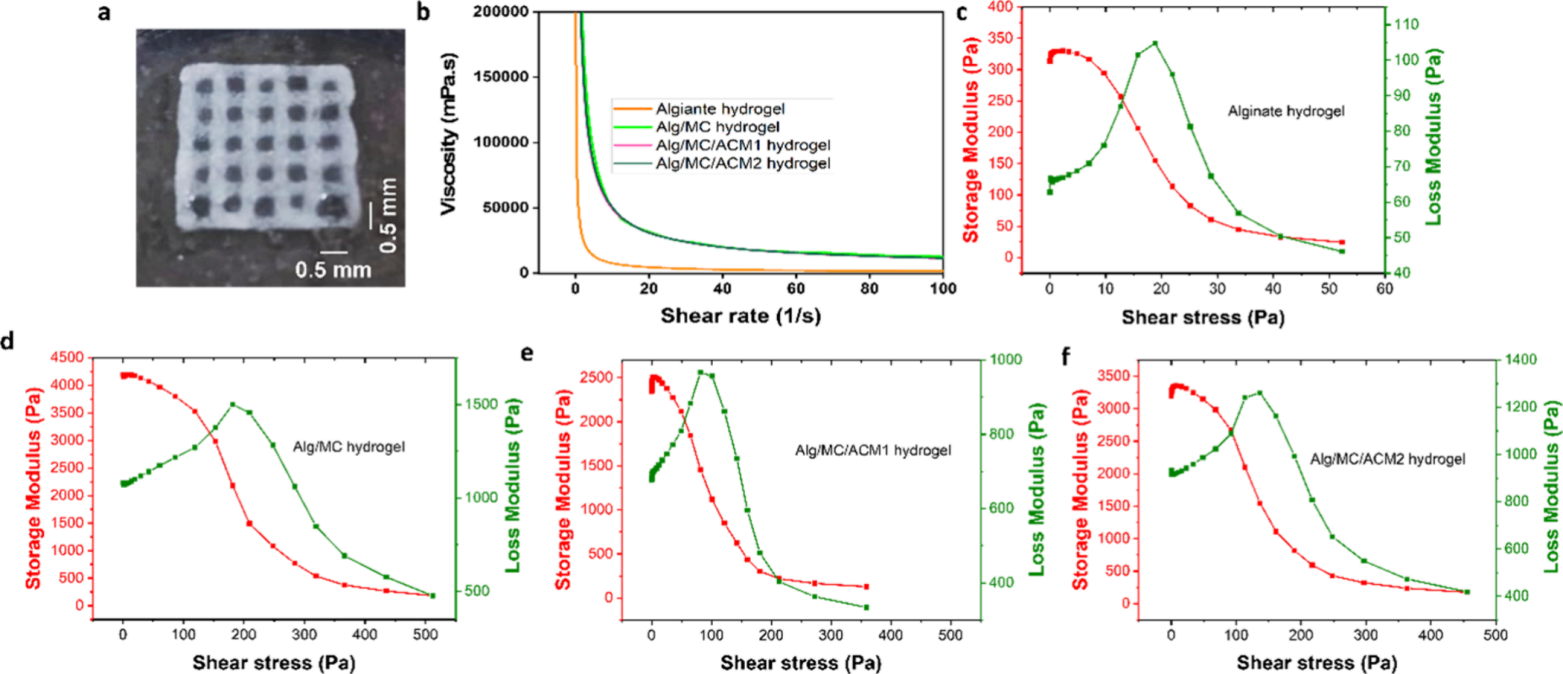
(a) Representative macroscopic image of 3D-bioprinted grid with a 10 × 10 × 1.2 mm dimension. Scale bar: 0.5 mm. Rheological analysis: (b) viscosity vs shear rate at 37 °C, and storage modulus and loss modulus vs shear stress of (c) alginate hydrogel, (d) Alg/MC, (e) Alg/MC/ACM1, and (f) Alg/MC/ACM2 hydrogels.

### 3.2 Rheological Characterization and Bioprinting

The rheological properties of the synthesized alginate, Alg/MC, and Alg/MC/ACM hydrogels were evaluated using a rheometer, as shown in Figure 3b−f. In the viscosity versus shear rate analysis (Figure 3b), it was observed that all hydrogels demonstrated shear-thinning behavior, i.e., there was a decrease in viscosity with increasing shear rate. The neat alginate hydrogel showed the least viscosity over 0−100 s^−1^ shear rate, which indicated its weak internal structure. In contrast, the Alg/MC, Alg/MC/ACM1, and Alg/MC/ACM2 hydrogels showed higher viscosity due to blending with MC. Further, we also observed that the addition of ACM did not have any effect on the viscosity of the Alg/MC hydrogel. Next, the viscoelastic properties of the hydrogels were examined. As shown in Figure 3c−f, all samples exhibited a predominantly elastic gel-like behavior, with *G*′ consistently higher than *G*″ across the initial stress range. In the alginate hydrogel (Figure 3c), a distinct crossover between *G*′ and *G*″ was observed at a shear stress of ∼10 Pa, where the transition from solid-like to liquid-like behavior occurred. For the Alg/MC hydrogel (Figure 3d), the storage modulus gradually declined with increasing shear stress, and a crossover was observed at ∼100 Pa. As observed with viscosity, the incorporation of ACM did not have any effect on the storage and loss modulus of the Alg/Mc hydrogel. Next, bioprinting was carried out using a Cellink Inkredible+ bioprinter. Alg/MC/ACM scaffold was bioprinted at an optimized pressure of 80 kPa with a 25 G nozzle. Grid-like scaffolds of dimension 10 × 10 × 1.2 mm were bioprinted as seen in Figure 3a.

### 3.3 Swelling and Degradation Analysis

To evaluate the swelling behavior, scaffolds were placed in PBS and incubated at 37 °C, and weights were recorded at different time points (Figure 4a). The neat alginate scaffold showed a lower swelling ratio when compared to Alg/MC, Alg/MC/ACM1, and Alg/MC/ACM2 scaffolds. Alg scaffolds showed a gradual increase in swelling over 0.5 to 24 h, reaching 77.55 ± 13.42. In contrast, Alg/MC, Alg/MC/ACM1, and Alg/MC/ACM2 scaffolds exhibited a rapid increase in swelling within the first 0.5 h, attaining 177.23 ± 4.91, 196.35 ± 26.18, and 193.79 ± 10.08%, respectively. At 24 h, the swelling ratios of Alg/MC, Alg/MC/ACM1, and Alg/MC/ACM2 scaffolds were 290.52 ± 12.35, 305.05 ± 21.49, and 303.31 ± 13.52, respectively. Further, the degradation rate of the scaffolds was studied. As seen in Figure 4b, all the scaffolds showed a degradation rate of ∼50−60% after 180 days of study. The degradation pattern remained the same across all four scaffolds.

**Figure 4:**
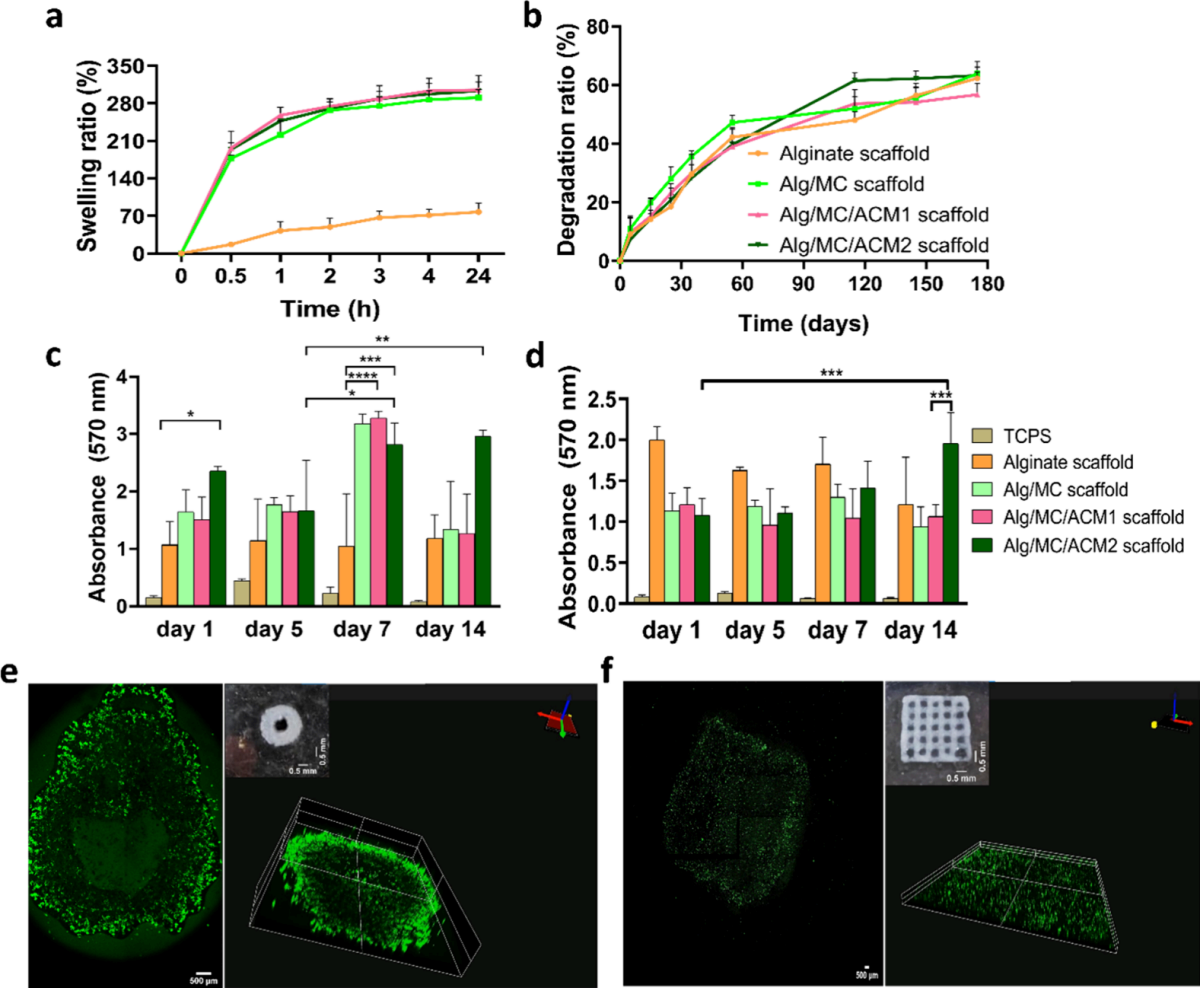
(a) Swelling analysis and (b) degradation analysis of alginate, Alg/MC, Alg/MC/ACM1, and Alg/MC/ACM2 scaffolds in PBS. MTT assay evaluating absorbance of (c) rat Schwann RSC96 cell and (d) PC12 cells on alginate, Alg/MC, Alg/MC/ACM1, and Alg/MC/ACM2 scaffolds (*n* = 3) * *p* < 0.05, ** *p* < 0.01 *** *p* < 0.001, and **** *p* < 0.0001. Data is represented as mean ± standard deviation. Statistical analysis was performed using two-way ANOVA followed by Tukey’s multiple comparison test. Representative confocal fluorescence images of Live/Dead assay of (e) four layer bioprinted cylindrical blood vessel-like structure and (f) three layer bioprinted grid structure using rat Schwann RSC96 cells at day 7. Scale bar: 500 μm.

### 3.4 Cell Viability and Proliferation

The biocompatibility of the scaffolds was evaluated by MTT assay using rat Schwann cells (Figure 4c) and PC12 cells (Figure 4d). As seen in Figure 4c, the proliferation of Schwann cells on the neat Alg scaffolds gradually decreased over a 14 day period, which indicated its poor bioactivity. Alg/MC and Alg/MC/ACM1 scaffolds showed similar behavior in cell proliferation at all day points. It was observed that there was a significant increase in proliferation in Alg/MC and Alg/MC/ACM1 scaffolds on day 7 when compared to other scaffolds, which declined on day 14. With the cell response that was observed in Alg/MC and Alg/MC/ACM1 scaffolds, it could be said that the presence of 1 μg/mL of ACM in the scaffold did not show any significant improvement in cell proliferation. However, in Alg/MC/ACM2 scaffolds, it was observed that there was a significant increase in proliferation on day 1, which reduced on day 5. On day 7, there was a significant increase in cell proliferation in Alg/MC/ACM2 scaffolds, and it was maintained through day 14. It could also be observed that on day 14, Alg/MC/ACM2 showed significantly high cell proliferation when compared to all other scaffolds.

Next, cell proliferation of PC12 cells was analyzed on the scaffolds (Figure 4d). On day 1, it was observed that Alg scaffolds showed increased cell proliferation in comparison to all other scaffolds. Alg scaffolds showed a gradual reduction in cell proliferation from day 1 to 14. Similar to the trend seen with Schwann cells, PC12 cells also showed the same behavior when cultured in Alg/MC and Alg/MC/ACM1 scaffolds over all the time points, indicating that 1 μg/mL of ACM did not have much impact on PC12 cell proliferation. In Alg/MC/ACM2 scaffolds, there was a gradual increase in cell proliferation from day 1 to 14. On day 14, Alg/MC/ACM2 showed significantly higher cell proliferation than all other scaffolds. Overall, with the cell proliferation results, we observed that 1 μg/mL of ACM did not show any difference when compared to scaffolds with no ACM. However, 10 μg/mL of ACM showed significantly improved cell proliferation in both Schwann cells and PC12 cells. Further, cell viability for Alg/MC/ACM2 scaffold was analyzed using Live/Dead assay (Figure 4e,f). It was observed that when cells were incorporated within the Alg/MC/ACM2 hydrogel, and bioprinting was carried out, the cells showed good viability post 7 days in culture. Even after 7 days in culture, the cells were observed to be encapsulated within the bioprinted structure, indicating that the pressure applied and the cross-linking strategy developed during bioprinting did not affect cell viability.

### 3.5 In Vivo Animal Studies

Further in vivo studies were carried out in the IAN crush injury model.^7,8^ The surgical approach used in the current work was controlled compression using forceps for 30 s. The Alg/MC/ACM2 scaffolds were implanted, and the animals were maintained for 21 days. Behavioral assessment was performed on day 21, comparing the injury control group and Alg/MC/ACM2 scaffold-treated group. The surgery was performed on the right side of the face of all of the animals. In the injury control group (Supporting Video S1, Supporting Information), mechanical stimulation of the injury (right) side elicited minimal or absent withdrawal responses, with reduced head or eye movement, indicating diminished mechanical sensitivity following IAN crush injury. In contrast, animals treated with the Alg/MC/ACM2 scaffolds (Supporting Video S2, Supporting Information) demonstrated clear head and eye movement, which was comparable to the contralateral side, suggesting improved sensory function.

Post 21 days, animals were humanely sacrificed, and the IAN nerve tissue was removed and fixed for histological evaluation. In the hematoxylin and eosin staining (Figure 5C), the control group showed no injury and no treatment, and the proper architecture of a peripheral nerve was seen. In injury control, disorganized tissue architecture was observed, indicating poor regeneration. In the Alg/MC scaffold group, a mild degree of cellular infiltration and the presence of fibrotic tissue were observed. In Alg/MC/ACM2 scaffolds, densely populated cells that are aligned longitudinally were observed, indicating initiation of the regeneration process. Further, Luxol Fast Blue staining was done to evaluate myelin sheath formation. In injury control, the absence of myelinated nerve fibers was observed, confirming that the crush injury that was performed was effective and there was minimal regeneration. In the Alg/MC group, there was an absence of blue or purple color, indicating no myelination in the tissue. In the Alg/MC/ACM2 scaffold group, a uniform blue coloration was observed across the tissue. Overall, the histological evaluation confirmed that the incorporation of Schwann cell ACM within the Alg/MC scaffold significantly enhanced IAN regeneration.

**Figure 5:**
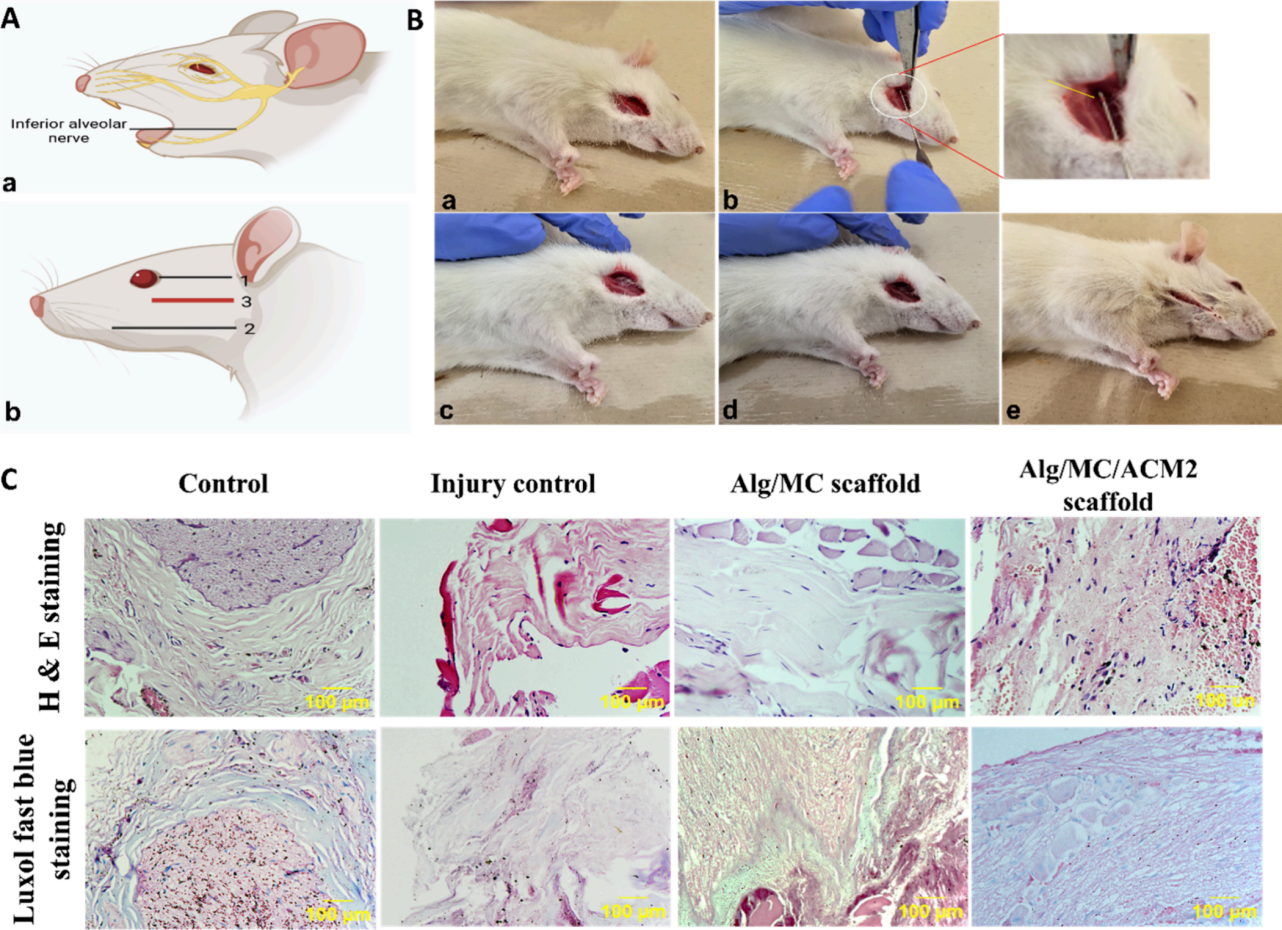
(A) Schematic representation of (a) inferior alveolar nerve in rat and (b) surgical landmarks 1: outer canthus of the eye to the base of the ear, 2: elongation of labial commissure, and 3: incision line. Created using BioRender.com. (B) Representative macroscopic images of surgical induction of IAN crush injury and scaffold implantation in the rat model. (a) Initial incision made at the posterior border of the mandible to access the IAN pathway. (b) Exposure of IAN. (c) Placement of Alg/MC/ACM2 printed grid structure at the crush injury site. (d) Approximation of muscle and (e) closure of the surgical site using Liquibond. (C) Histological evaluation of IAN tissue in different experimental groups using hematoxylin and eosin (H&E) staining and Luxol fast blue staining. Scale bar: 100 μm.

## 4 Discussion

Inferior alveolar nerve injury is a clinically significant complication associated with various dental and maxillofacial procedures, including mandibular third molar extraction, dental implant placement, orthognathic surgery, and mandibular fractures.^7,31–33^ The IAN is the largest terminal branch of the mandibular division of the trigeminal nerve and is responsible for sensory innervation of the lower lip, chin, and mandibular teeth. Injury to this nerve can lead to neurosensory disturbances such as paraesthesia, hypoesthesia, or dysesthesia in the affected regions, which may adversely impact essential functions such as speaking, chewing, and overall quality of life.^4,7,11^ In clinical settings, the incidence of IAN injury varies depending on the procedure performed; for example, mandibular third molar extraction has been reported to cause nerve injury in approximately 0.35−8.4% of cases.^34^ These injuries may occur due to direct trauma to the nerve, compression caused by hematoma or displaced structures, or mechanical disruption during surgical manipulation within the mandibular canal. Given the functional and psychosocial implications associated with IAN injury, the development of regenerative strategies, including biomaterial-based scaffolds, has gained considerable attention to promote nerve regeneration. Among the approaches of fabricating scaffolds, 3D bioprinting has emerged as a promising strategy with precise control over architecture, porosity, and spatial distribution of bioactive components. Bioprinted scaffolds can mimic the native extracellular matrix and provide a conducive microenvironment that supports Schwann cell adhesion, proliferation, and neurotrophic factor secretion, thereby facilitating axonal regeneration and directional neurite outgrowth.^35^ The ACM-based hydrogel developed in this study provides both biochemical cues and structural support, which play an important role in peripheral nerve regeneration. Extracellular matrix-derived materials are known to contain multiple proteins that regulate cell adhesion, migration, and differentiation.^23,36^ The detection of 904 proteins within ACM in our study suggests that multiple signaling pathways may contribute to the neuroregenerative effects. In our study, we did not identify specific active components for nerve regeneration. However, a study by Fine et al.^37^ suggests neurotrophic factors such as nerve growth factor and brain-derived neurotrophic factor have been shown to promote neuronal differentiation and improve nerve regeneration outcomes in vivo. In addition, Song et al.^38^ described hydrogel-based scaffolds incorporating NGF and BDNF, which demonstrated improved nerve fiber regrowth and functional recovery. In one of our previous works, Nagarajan et al.^23^ demonstrated that the incorporation of Schwann cell−derived acellular matrix provides essential biochemical cues that support neuronal survival and enhance nerve regeneration. The study showed that ACM-coated scaffolds significantly promote remyelination by upregulation of myelin-associated markers such as PMP22. Additionally, the multifunctional scaffold incorporating ACM exhibited both antioxidant and promyelinating effects, highlighting its role in creating a proregenerative microenvironment for nerve regeneration. Based on the studies mentioned above, we hypothesized that ACM may provide superior regenerative potential by delivering multiple bioactive cues simultaneously. To this end, we developed Alg/MC/ACM scaffolds with two concentrations to understand the effect of ACM on peripheral nerve regeneration and compared it with alginate and Alg/MC scaffolds. Free ACM injection was not included because: ACM in solution form is rapidly diffused and cleared in vivo, the confined bony canal of the IAN limits retention of injectable biomolecules, and sustained localized release of ACM was a central rationale of this scaffold. Alg/MC hydrogel was optimized, and bioprinting was done as mentioned in our previous literature.^24^ In a study by Silva et al.,^39^ 15 μL of human adipose stem cell-derived secretome was reported to effectively reduce inflammation and promote proregenerative processes in a spinal cord injury model. However, we recognized that the addition of ACM in μL could alter the overall protein concentration. Therefore, to ensure uniformity across all groups, the concentrations were optimized in μg. Based on this evidence and understanding, two concentrations of ACM (1 and 10 μg/mL) were selected in our study to preliminarily evaluate its regenerative potential. Also, ACM-based approaches have not yet been extensively explored for peripheral nerve regeneration. This approach provided an initial assessment of dose-dependent effects, while more extensive dose−response studies will be explored in future studies.

Further, rheological studies were carried out to analyze the effect of the addition of ACM on viscosity. Rheological properties play a critical role in determining the suitability of hydrogels for extrusion-based 3D bioprinting. A key characteristic of printable bioinks is shear-thinning behavior, where viscosity decreases with increasing shear rate. This property facilitates extrusion through narrow printing nozzles under applied pressure while allowing the material to rapidly recover its viscosity once the shear stress is removed.^40,41^ All hydrogel formulations in our study exhibited shear-thinning characteristics. Alginate-based hydrogels have been extensively used in bioprinting because their rheological behavior enables controlled flow through printing nozzles while maintaining structural integrity after deposition. The presence of methylcellulose further contributed to the viscosity and stability of the hydrogel system. Amplitude sweep analysis further provided insight into the viscoelastic properties of the hydrogels by measuring the storage modulus (*G*′) and loss modulus (*G*″). In general, a higher storage modulus compared with loss modulus indicates that the material behaves as a stable elastic gel capable of maintaining structural integrity. In the present study, neat alginate hydrogel exhibited an early crossover between *G*′ and *G*″ at relatively low shear stress, indicating that the hydrogel loses its structural integrity under minimal mechanical stress. In contrast, the Alg/MC hydrogel formulation demonstrated a delayed crossover point, suggesting improved resistance to deformation under higher shear stress. This indicates that the composite hydrogel can withstand greater mechanical stress during extrusion, thereby improving printing stability. Following the crossover point, the loss modulus became greater than the storage modulus, indicating a transition from elastic to viscous behavior. This behavior suggests that the hydrogel may temporarily lose structural rigidity during extrusion but can recover once the applied stress is removed. However, increased viscous behavior may compromise the structural stability of printed constructs. Therefore, to enhance shape fidelity and maintain the geometry of the printed scaffolds, postprinting ionic cross-linking using 4% calcium chloride was performed. Ionic cross-linking of alginate through calcium ions is a well-established strategy for stabilizing printed structures and improving the mechanical integrity of bioprinted scaffolds. It was also observed that the incorporation of ACM did not affect the rheological properties of Alg/MC hydrogel. Overall, the rheological analyses confirmed that all hydrogel formulations exhibit shear-thinning and viscoelastic properties required for bioprinting.

Swelling analysis also revealed that the incorporation of ACM within the Alg/MC hydrogel did not significantly alter the swelling properties of the scaffolds. The comparable swelling ratios observed for the Alg/MC, Alg/MC/ACM1, and Alg/MC/ACM2 scaffolds suggest that the addition of ACM did not interfere with the hydrogel network structure or water uptake capacity. This observation is beneficial for tissue engineering applications as maintaining consistent swelling behavior ensures adequate diffusion of nutrients and oxygen within the scaffold microenvironment.^42^ It is also mentioned by Obeng et al.^43^ that swelling in the hydrogel also gives it a soft tissue-like mimicry for integration with tissues. Similarly, degradation studies demonstrated that all scaffold groups exhibited comparable degradation profiles. It was observed that alginate scaffolds showed a similar degradation rate as Alg/MC, Alg/MC/ACM1, and Alg/MC/ACM2 (Figure 4b). This could be due to the two-step cross-linking using calcium chloride that was employed. In this study, all of the scaffolds exhibited an initial weight loss within the first 5 days of incubation in PBS; therefore, day 5 was considered as day 0 for calculating the degradation ratio over the 180 day study period.

To evaluate the biological performance of the scaffolds, in vitro cytocompatibility studies were performed using rat Schwann cells (RSC96) and PC12 neuronal cells. Schwann cells were selected because they are the primary glial cells of the peripheral nervous system and play a crucial role in nerve regeneration through myelination, secretion of neurotrophic factors, and guidance of axonal regrowth.^44–47^ PC12 cells, derived from rat pheochromocytoma, are widely used as a neuronal model because they exhibit neuronal differentiation and neurite outgrowth in response to extracellular cues. Previous studies have extensively used both cell types to evaluate biomaterials intended for peripheral nerve regeneration.^45,48,49^ The proliferation behavior of Schwann cells observed in this study demonstrated an initial adaptation phase followed by increased proliferation in the ACM-containing scaffold group. The reduced proliferation observed during the early culture period reflects the cellular adaptation to the newly formed three-dimensional microenvironment. Such adaptation periods of approximately 3−5 days have been reported in previous studies involving cells encapsulated within hydrogel matrices.^50^ The MTT results obtained with PC12 cells further supported the bioactivity of the ACM-containing scaffolds. While initial proliferation was higher in the alginate control group, a gradual decline was observed over time. Alginate initially permits surface cell attachment and metabolic activity. However, due to a lack of adhesion motifs, there is a decrease in long-term cell proliferation.^51–53^ In contrast, the Alg/MC/ACM2 scaffold demonstrated a progressive increase in PC12 cell proliferation from day 1 to day 14. This trend suggests that the presence of Schwann cell-derived ACM provided bioactive cues that supported neuronal cell survival and proliferation. Similar findings have been reported in the literature where bioactive extracellular matrix components were incorporated into hydrogels to enhance neuronal cell proliferation and differentiation.^37,38,54,55^

The IAN crush injury model serves as a clinically relevant in vivo system for studying peripheral nerve regeneration in the craniofacial region. The crush injury model closely mimics common clinical conditions such as trauma during mandibular surgeries or local anesthesia administration, where axonal continuity is partially disrupted, leading to demyelination, thereby allowing for reproducible axonal damage.^14^ As seen in in vitro cell proliferation studies, we observed that Alg/MC/ACM2 scaffolds showed significant results in cell proliferation of both Schwann cells and PC12 cells. Hence, for the in vivo study, the Alg/MC/ACM2 scaffolds were utilized. Additionally, alginate scaffolds alone did not show a substantial enhancement in cell proliferation in the in vitro studies. Therefore, on the basis of these preliminary findings, only the most promising formulation (Alg/MC/ACM2) was selected for the in vivo evaluation. Furthermore, to minimize the number of experimental animals used and adhere to ethical considerations in animal experimentation, additional groups were not included in the in vivo studies. Implantation of the Alg/MC/ACM2 bioprinted scaffold at the crush site aimed to create a localized microenvironment that would facilitate axonal regeneration, myelination, and functional recovery. Functional recovery was assessed by using mechanical stimulation of the facial region. Although mechanical sensitivity is ideally quantified using von Frey filaments, their unavailability necessitated the use of a brush to evoke the responses. Despite this limitation, the observed behavioral responses suggested improved sensory recovery in animals treated with the ACM-containing scaffold compared to injury controls. Improved behavioral studies and electrophysiological assessments, such as nerve conduction velocity, would provide a more comprehensive functional evaluation. Because of technical constraints and the anatomical confinement of the rat IAN, electrophysiological recording was not performed in this study. However, incorporating such assessments will be considered in future studies to enable a more comprehensive evaluation of the functional nerve recovery. Histological evaluation using hematoxylin and eosin staining provided further insights into the tissue morphology, cellular infiltration, and inflammatory response at the injury site. The presence of organized cellular structures and aligned cells in the scaffold-treated group indicates the initiation of regenerative processes within injured nerve tissue. Such a structural organization is typically associated with Schwann cell migration and axonal guidance during peripheral nerve regeneration.^56^

## 5 Conclusions

The inferior alveolar nerve crush injury represents a persistent clinical challenge mainly due to limited effective regenerative strategies. Current treatment protocols involving drugs often focus on symptomatic treatment and fail to restore function and regeneration. To offer better treatment options, we developed bioprinted scaffolds composed of alginate, methylcellulose, and Schwann cell-derived acellular matrix. In the present work, we incorporated ACM within Alg/MC hydrogel in two concentrations (1 and 10 μg/mL) to determine the minimal effective dose capable of eliciting regenerative effects while maintaining the stability of the hydrogel under standard storage conditions. The incorporation of ACM did not alter the rheological properties of Alg/MC hydrogel, and good printability and shape fidelity were observed. The blending of 1 μg/mL ACM did not show any comparable results to Alg/MC hydrogel in terms of cell proliferation and viability. However, the addition of 10 μg/mL of ACM (Alg/MC/ACM2) showed significant improvement in cell proliferation and viability. Further, an in vivo IAN crush injury model in SD rats was developed to demonstrate the effect of Alg/MC/ACM2 scaffolds. The results suggest that Alg/MC/ACM2 scaffolds showed good tissue regeneration and enhanced myelination when compared to injury and Alg/MC control groups. Collectively, the results demonstrated that Alg/MC/ACM bioprinted scaffolds offered a promising bioactive platform for promoting IAN regeneration.

## Supplementary Material









## Data Availability

The data supporting this article have been included as part of the Supporting Information file.

## Notes

Supplementary data sheet containing Tables S1−S4 (XLSX)
Mechanical stimulation of the injury (right) side elicited minimal or absent withdrawal responses, with reduced head or eye movement, indicating diminished mechanical sensitivity following IAN crush injury (MP4)
Animals treated with the Alg/MC/ACM2 scaffolds demonstrating clear head and eye movement, which was comparable to the contralateral side, suggesting improved sensory function (MP4)
Materials information and LC/MS methodology (PDF)
