# Supplementary material for: Development of Acellular Matrix-Based Bioprinted Scaffold for Inferior Alveolar Nerve Regeneration
Source: ACS Omega. 2026 Jun 11;11(24):35467–78. doi: 10.1021/acsomega.6c01059 (PMC13294912; doi:10.1021/acsomega.6c01059)
Supplement: Supplementary file 4 [file ao6c01059_si_004.pdf]

Development of Acellular Matrix based Bioprinted Scaffold for Inferior Alveolar Nerve Regeneration

Nasera Rizwana,[a] Kaustubh Raundal,[a] Yogesh H S,[b] Radhika Kawathe,[c,d] Sridhar Chinthakindi, [c,d] Rohit Kumar Sarda[e], Ashwath Acharya,[f] Manasa Nune[a*]

[a]*Manipal Institute of Regenerative Medicine, Manipal Academy of Higher Education, Manipal 576104, Karnataka, India*

[b]*Department of Pharmacology, NITTE College of Pharmaceutical Sciences (NITTE Deemed to be University), Bangalore Campus, Karnataka, India*

[c]*Manipal Academy of Higher Education, Manipal, Karnataka, 576104, India*

[d]*Institute of Bioinformatics, Discoverer Building, 7th Floor, International Tech Park, Whitefield, Bangalore - 560 066, Karnataka, India*

[e]*Department of Pathology, Sikkim Manipal Institute of Medical Sciences, Sikkim Manipal University, Tadong, Gangtok, Sikkim 737102, India*

[f]*Department of Hand Surgery, Kasturba Medical College, Manipal, Centre for Congenital Hand Differences, Manipal 576104, Karnataka, India*

*Corresponding author: manasa.nune@manipal.edu

Supporting Information

1. Materials

Sodium alginate (CAS: 9005-38-3) and phosphate buffer saline (PBS) tablets (P4417) were purchased from Sigma-Aldrich (India). Dimethyl sulfoxide (MB058) was purchased from HiMedia Laboratories Pvt Ltd (India). Methylcellulose was purchased from TCI. Calcium chloride (CAS: 10035-04-8) was purchased from SRL chemicals. Rat Schwann cells (RSC-96) were obtained from American Type Culture Collection (CRL-2765 ATCC, USA). PC12 was obtained from National Centre for Cell Sciences, Pune. Fetal bovine serum (FBS) (lot no 2440096), antibiotics penicillin-streptomycin (P4333), antibiotic-antimycotic (A5955), and Dulbecco's eagle medium (DMEM) (lot no. 2323533) were purchased from Gibco (India). MTT kit (TC191-1G) was purchased from HiMedia Laboratories Pvt Ltd (India). Live/dead kit (lot no. 2015555) was purchased from Invitrogen (USA). Urea, iodoacetamide (IAA) and trifluoroacetic acid (TFA) were purchased from Sigma-Aldrich (St. Louis, MO, USA). Triethylammonium bicarbonate (TEAB) buffer and dithiothreitol (DTT) were obtained from Thermo Fisher Scientific. Sequencing-grade modified trypsin was obtained from Worthington Biochemical Corporation (Lakewood, NJ, USA). Sep-Pak C18 cartridges were procured from Waters Corporation (Milford, MA, USA). Acetone and formic acid were purchased from Honeywell (Charlotte, NC, USA). LC–MS grade acetonitrile and water were obtained from J.T. Baker (Phillipsburg, NJ, USA). Surgical glue was purchased from Liquiband, Optima.

2. LC-MS/MS Analysis

The protein digested samples were analyzed on a Q-Exactive™ plus Biopharma mass spectrometer (Thermo Scientific, Sunnyvale, CA, USA) coupled to an Easy-nano-UPLC system (Thermo Fisher Scientific, Carlsbad, CA, USA). Peptides were separated on a PepMap RSLC C18 column (50cm x 100A, 2.0µm; Thermo Scientific), maintained at 30 °C with an Acclaim PepMap 100 nanoviper (100 µm x 2 cm) precolumn. We used 0.1% formic acid in water (mobile phase A) and 0.1% formic acid in 85:15 (acetonitrile: water) (mobile phase B) (Honeywell Charlotte, NC, USA). Gradient elution was performed with the following protocol: 0–5.0 min, 2-5% B; 5.0–55 min, 5–15% B; 55–75 min, 15%–45% B; 75-85 min, 45%-95% B hold for 10 min, followed by equilibration for next run for 5 min at 2% B. The flow rate was 300 nL/min, and the injection volume was 12 µL. A full MS-ddMS2 (Full-scan data-dependent MS/MS) scan mode with a higher-energy collisional dissociation (HCD) method was used. The equipment was operated with a control software, Xcalibur, version 4.2.28.14. The Full scan MS parameters were set as follows: scan range, 350 to 2000 m/z; resolution, 70,000; AGC target, 1×10^6; maximum injection time (IT), 60 ms. The parameters of dd-MS2 were as follows: resolution, 17,500; AGC target, 2×10^5; maximum IT, 120 ms; loop count, 15; isolation window, 1.2 m/z. The normalised collision energy (NCE) was set at 27 V. The following parameters were used for the heated electrospray ionization source (HESI source): spray voltage, 3.5 kV for ESI+; sheath gas flow rate, 35 arbs; auxiliary gas flow rate, 25 arbs; sweep gas flow rate, 0 arb; capillary temperature, 325°C; s-lens RF level, 55; and probe heater temperature, 300 °C.[1,2]

References

(1) Garapati, K.; Budhraja, R.; Saraswat, M.; Kim, J.; Joshi, N.; Sachdeva, G. S.; Jain, A.; Ligezka, A. N.; Radenkovic, S.; Ramarajan, M. G.; Udainiya, S.; Raymond, K.; He, M.; Lam, C.; Larson, A.; Edmondson, A. C.; Sarafoglou, K.; Larson, N. B.; Freeze, H. H.; Schultz, M. J.; Kozicz, T.; Morava, E.; Pandey, A. A Complement C4-Derived Glycopeptide Is a Biomarker for PMM2-CDG. *JCI Insight* **2024**, *9* (7), e172509. https://doi.org/10.1172/jci.insight.172509.

(2) Jain, A. P.; Ghose, V.; Munshi, S.; Bhat, F. A.; Dey, G.; Nanjappa, V. Mass Spectrometry-Based Proteomic Analysis to Characterize Cisplatin Induced Early Signaling Events in Head and Neck Squamous Cell Carcinoma. *Mol Cell Oncol* **2024**, *11* (1), 2328873. https://doi.org/10.1080/23723556.2024.2328873.
